# Rediscovery of the sun bear (*Helarctos malayanus*) in Yingjiang County, Yunnan Province, China

**DOI:** 10.24272/j.issn.2095-8137.2017.044

**Published:** 2017-07-18

**Authors:** Fei Li, Xi Zheng, Xue-Long Jiang, Bosco Pui Lok Chan

**Affiliations:** ^1^Kadoorie Conservation China, Kadoorie Farm & Botanic Garden, Hong Kong 999077, China.; ^2^State Key Laboratory of Genetic Resources and Evolution, Kunming Institute of Zoology, Chinese Academy of Sciences, Kunming Yunnan 650223, China

## DEAR EDITOR,

The sun bear, *Helarctos malayanus* (Raffles, 1821), is a forest-dependent bear species distributed in tropical Southeast Asia. The species was previously reported from scattered localities in southwestern China, which is at the northeastern edge of its global range. Due to the scarcity of reliable recent records, some authorities cast doubt on the continued existence of sun bear in China. Here we present the rediscovery of this species in Yingjiang County, western Yunnan Province, China, near the international border with Myanmar's Kachin State.

The sun bear, *Helarctos malayanus* (Raffles, 1821), is the rarest species in the family Ursidae in China, and is listed as a Category Ⅰ species on the National Key Protected Animal List. The latest Red List of China's Vertebrates (Jiang et al., 2016) evaluated the sun bear as Critically Endangered (CR), though globally it is categorized as Vulnerable (VU) species by the International Union for Conservation of Nature (IUCN) Red List, indicating the species has undergone a suspected > 30% decline in the global population (Fredriksson et al., 2008).

Globally, the sun bear occurs in northeast India, Bangladesh, and throughout Southeast Asia including the islands of Sumatra and Borneo. The sun bear is the most arboreal of all bear species and is found predominantly in lowland dipterocarp rainforest (Smith & Xie, 2008). Due to habitat destruction and poaching for their body parts as traditional medicine, the sun bear is now extinct in Singapore (Fredriksson et al., 2008) and has possibly become extinct more recently in Bangladesh (Islam et al., 2010). Studies in Vietnam (Cano & Tellería, 2013), Borneo (Meijaard, 1999) and Sumatra (Wong et al., 2013) also reported declines in both abundance and distribution, and the species has been extirpated from much of its former range.

Literature on the presence of sun bear in China is scanty, and the IUCN Red List stipulated that its current distribution in China is unknown. Richard Lydekker (1849–1915), a British naturalist, was the first to report the occurrence of sun bear in the Tibetan area of China. However, his specimen of two skulls and a skin of unknown provenance came from a wildlife trader (i.e., Rowland Ward Ltd.), and the author pointed out that the skin has external features of an Asiatic black bear (*Ursus thibetanus*) rather than that of a sun bear (Lydekker, 1906). Ernest Henry Wilson (1876–1930), another English who spent 11 years exploring southwestern China collecting plants, subsequently analyzed the trade routes and patterns of wildlife products of the area, and expressed his skepticism about the provenance for Lydekker's specimen, and argued that the skulls probably originated from the warmer regions of Yunnan Province while the skin was that of a Asiatic Black Bear (Wilson, 1913).

The first unequivocal record of sun bear occurrence in southern Yunnan came from Wang (1987), who collected a female specimen from the Red River Basin in 1972. Yin & Liu (1993) reported the collection of two sun bear specimens from Tibet during 1987–1990, and reported that sun bears occurred at an altitudinal range of 3 000-3 500 m a.s.l. in Mangkang County. It is of note that the highest known elevational record for sun bear is at 2 143 m a.s.l. in Sumatra (Fredriksson et al., 2008), the Tibet records thus warrant some investigation. Ma et al. (1994) and Hu (1995) reported the capture of a sun bear in Jingxi County of Guangxi Province, by the Sino-Vietnamese border, but these authors did not provide detailed information of this record. A number of publications reported the existence of sun bears in Sichuan Province and the northwestern part of Yunnan, but these reports are not supported by solid evidence such as specimens or photos (Jiang et al., 2015; Shi & Zhao, 1982; Smith & Xie, 2008). The current status and distribution of sun bear in China is unknown though it was listed in recent publications (Pan et al., 2007; Wang, 2003), and some scientists suspected the species may already be extinct in China (Smith & Xie, 2008).

At 1927h on 23 October 2016, we obtained a 10 sec video footage of a bear species by a camera trap installed in a community forest in Yingjiang County, Dehong Dai and Jingpo Autonomous Prefecture, Yunnan Province (Supplementary Video, available online). Despite the poor light, we could clearly identify the subject animal as a sun bear with the following diagnostic features: head broad with a short snout; muzzle very short and pale in color; face pale in contrast to the black body; ears set low on sides of head, very small and rounded without ear tuft; coat black, very short and dense; crescent-shaped pale-colored chest mark; limbs relatively slender and long, forelimbs bowed, forefeet turned inward.

The site of discovery is a disturbed montane rainforest at 1 000 m a.s.l. at N24°32', E97°34', adjacent to Tongbiguan Provincial Nature Reserve (Tongbiguan NR) less than 1 km from the international border with Kachin State of Myanmar. Camera traps from the same locality recorded several sympatric mammal species, including wild boar (*Sus scrofa*), red muntjac (*Muntiacus vaginalis*), Chinese serow (*Capricornis milneedwardsii*) and yellow-throated marten (*Martes flavigula*). We also camera-trapped the Asiatic black bear approximately 1.8 km east of the sun bear site in the same forest block, suggesting the two ursid species are sympatric in the western part of Yingjiang County.

Our sun bear record from Yingjiang produced the first image of the species for China, and represents a rediscovery of this species in Yunnan after an absence of 45 years. Although the site of discovery is very close to the boundary of Tongbiguan NR, the community forest is subject to high human disturbances, and under threats from being cleared for agriculture, as well as hydro-dam and road construction. We urge relevant government agencies to reconsider the necessity of all development plans of the general area to avoid further forest degradation, and step up protection and restoration efforts of natural forest surrounding Tongbiguan NR to reconnect fragmented lowland forest blocks, so as to enhance the future survival of tropical wildlife such as the highly threatened sun bear.

## ACKNOWLEDGEMENTS

We would like to express our gratitude to the Yingjiang County Propaganda Department and the Forestry Bureau, and local villagers for their hospitality, encouragement and support. We thank Quan Li of Kunming Institute of Zoology, Chinese Academy of Sciences, for checking bear specimens in his institution's collection, Will Duckworth and Lorraine Scotson for verification of sun bear identification.
